# Efficacy of Aflatoxin B1 and Fumonisin B1 Adsorption by Maize, Wheat, and Oat Bran

**DOI:** 10.3390/toxins16070288

**Published:** 2024-06-25

**Authors:** Youngsun Lee, Jenna M. Lemmetty, Hanna Nihtilä, Hanna Koivula, Serge Samandoulougou, Hagretou Sawadogo-Lingani, Kati Katina, Ndegwa H. Maina

**Affiliations:** 1 Department of Food and Nutrition, University of Helsinki, 00014 Helsinki, Finland; youngsun.lee@helsinki.fi (Y.L.); jenna.lemmetty@helsinki.fi (J.M.L.); hannanihtila@gmail.com (H.N.); hanna.m.koivula@helsinki.fi (H.K.); kati.katina@helsinki.fi (K.K.); 2 Institut de Recherche en Sciences Appliquées et Technologies/Département Technologie Alimentaire (IRSAT/DTA), Ouagadougou BP 7047, Burkina Faso; sserge1rech@gmail.com (S.S.); hagretou@yahoo.fr (H.S.-L.)

**Keywords:** aflatoxin B1, fumonisin B1, mycotoxin, bran, pericarp, adsorption

## Abstract

Mycotoxins, especially aflatoxin B1 (AFB1) and fumonisin B1 (FMB1), are common contaminants in cereal-based foods. Instances of contamination are predicted to increase due to the current challenges induced by climate change. Despite the health benefits of whole grains, the presence of mycotoxins in bran remains a concern. Nonetheless, previous research indicates that wheat bran can adsorb mutagens. Therefore, this study investigated the capacity of maize, wheat, and oat brans to adsorb AFB1 and FMB1 under varying *in vitro* conditions, including pH, binding time, temperature, particle size, and the amount of bran utilized. Maize bran demonstrated a high AFB1 adsorption capacity (>78%) compared to wheat and oat brans. However, FMB1 was not adsorbed by the brans, possibly due to its hydrophilic nature. Lower temperature (≤25 °C) enhanced AFB1 adsorption efficacy in wheat and oat bran, while for maize bran, the highest adsorption occurred at 37 °C. A linear model following Henry’s law best explained AFB1 adsorption by the brans. Further studies identified the pericarp layer of bran as the primary site of AFB1 adsorption, with the initial liquid volume being a critical factor. The study concludes that bran could potentially act as an effective bioadsorbent. Further research is essential to confirm the adsorption efficacy and the bioavailability of AFB1 through *in vivo* experiments.

## 1. Introduction

Mycotoxins are secondary metabolites produced by fungi such as *Aspergillus*, *Fusarium*, and *Penicillium* and are known to contaminate food products during primary production and storage [1,2]. Among hundreds of mycotoxins, aflatoxin B1 (AFB1), fumonisin B1 (FMB1), and deoxynivalenol (DON) are common mycotoxin contaminants in cereals and are associated with health issues including carcinogenicity, hepatotoxicity, and immunotoxicity [3]. According to the International Agency for Research on Cancer (IARC) classification, AFB1 is a well-known human carcinogen and FMB1 is classified as a possible carcinogen to humans [4,5]. Therefore, AFB1 is regulated with higher priority in 61 countries for single AFB1 and 76 countries for total aflatoxins [6].

Currently, unprecedented climate conditions, such as heavy precipitation, droughts, and other extreme weather conditions, continue to occur worldwide due to climate change. These changes are predicted to expand the habitats of pests and subsequently increase fungal infections due to enhanced insect damage, which renders crops more susceptible to mold infection and facilitates the transfer of fungal spores [7,8]. Consequently, climate change can affect mycotoxin production directly and indirectly, leading to shifts in the geographical distribution, patterns, and severity of mycotoxin occurrences [7]. Warmer weather conditions favor the growth of thermotolerant fungal species, potentially increasing the prevalence of *Aspergillus* over *Penicillium* species and, as a result, causing a rise in mycotoxin (e.g., aflatoxins) contamination in areas where it was previously rare. This phenomenon poses a significant threat to both food security and safety [9,10,11]. 

Whole grains and products containing whole grain flour are frequently recommended as part of a healthy diet because they retain all components of the grain—the bran, germ, and endosperm—providing more fiber, vitamins, minerals, and phytochemicals than refined grains. However, whole grains are unfortunately also associated with a high potential for mycotoxin contamination, which can be concentrated in the outer layer (bran) [12]. The bran layer of cereal grains, encompassing the pericarp, testa (seed coat), and aleurone layer [13,14], contributes significantly to the nutritional and health-promoting qualities of whole grains. These benefits include fecal bulking, increased satiety, and a reduced risk of colorectal cancer [14]. Furthermore, studies have also shown that cereal brans can bind hydrophobic mutagens such as 1,8-dinitropyrene, benzo[a]pyrene, and heterocyclic amines [15,16,17]. Therefore, it is highly plausible that the bran can also interact with mycotoxins. Consequently, an elucidation of the mechanisms underlying the interaction between bran and mycotoxins, along with its impact on their bioavailability of mycotoxins, is needed. 

Previous studies have indicated that plant fibers such as cellulose, hemicellulose, pectin, and lignin, as well as agricultural by-products such as wheat straw, almond hull, wheat offal, and micronized wheat fiber, have potential to bind mycotoxins [18,19,20,21,22,23]. Greco et al. [18] reported the adsorption of five mycotoxins including AFB1, FMB1, DON, zearalenone (ZEA), and ochratoxin A (OTA) by various agricultural by-products, concluding that mycotoxin adsorption varies among these by-products due to differences in material composition and surface properties. Specifically, grape by-products, artichoke, and almond by-products exhibited high efficacy in mycotoxin adsorption. For instance, wheat straw at 1 mg/mL dosage adsorbed 21% of AFB1, 6% of FMB1, 2% of DON, 23% of ZEA, and 0% of OTA at pH 7 with a toxin concentration of 1 μg/mL. Avantaggiato et al. [19] showed that a red grape pomace (5 mg/mL dosage) can adsorb AFB1 (82%), FMB1 (35%), ZEA (67%), and OTA (61%) at pH 7. Carson and Smith [20] indicated that lignin could mitigate feed refusal and growth depression in rats exposed to the T-2 toxin. Aoudia et al. [21] suggested that micronized wheat fiber can reduce the bioavailability of OTA in piglets, as evidenced by decreased weight loss and reduced OTA levels in plasma, kidney, and liver. Moreover, Frape et al. [22,23] reported that wheat offal (bran) can reduce the toxicity of AFB1, as demonstrated by reduced incidence of tumors and increased excretion of AFB1 in feces. Despite this potential, the capacity of bran to bind mycotoxins has not been systematically investigated. In this study, we evaluated the ability of oat, wheat, and maize brans to adsorb AFB1 and FMB1 effectively and also investigated factors affecting the adsorption of AFB1 by brans, including binding time, temperature, bran amount, pH, and solvent volume. Additionally, the primary adsorption site in bran was examined to gain a better understanding of the interaction between bran and AFB1. 

## 2. Results and Discussion

### 2.1. Adsorption of AFB1 and FMB1 by Brans

**Figure 1:**
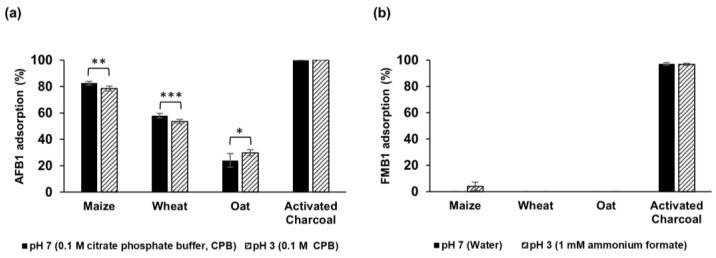
AFB1 ((**a**), 1 μg/mL) and FMB1 ((**b**), 5 μg/mL) adsorption by maize, wheat, and oat bran (1.7% *w*/*v*) at different pH values. The presented data are the mean ± standard deviation from three replicates. Statistical significance is denoted by asterisks (*** *p* < 0.001, ** *p* < 0.01, and * *p* < 0.05).

All three brans adsorbed AFB1 but none of them showed any adsorption capacity for FMB1. At 1.7% bran concentration, 25 °C, and 90 min binding time, maize bran adsorbed 82.5% and 78.5%, wheat 57.9% and 53.4%, and oat 24.0% and 29.8% at pH 7 and 3, respectively (Figure 1). The positive control using activated charcoal showed 100% and 97% AFB1 and FMB1 adsorption, respectively. This result is consistent with previous studies that utilized activated charcoal as an effective adsorbent for mycotoxins [24,25] and it confirmed that the experimental procedures used here were appropriate. 

The different adsorption capacities of AFB1 and FMB1 by bran can be attributed to the hydrophobicity of AFB1 and hydrophilicity of FMB1. Furthermore, the predicted pKa 3.16 [26] for carboxylic groups of FMB1 indicated that they are ionized at pH 7, thus favoring more interaction with water. Similarly, previous studies have indicated that bran binds hydrophobic mutagens such as 1,8-dinitropyrene, heterocyclic amines, and polycyclic aromatic hydrocarbons [15,16,17]. 

### 2.2. Effects of Bran Amount and pH on AFB1 Adsorption

**Figure 2:**
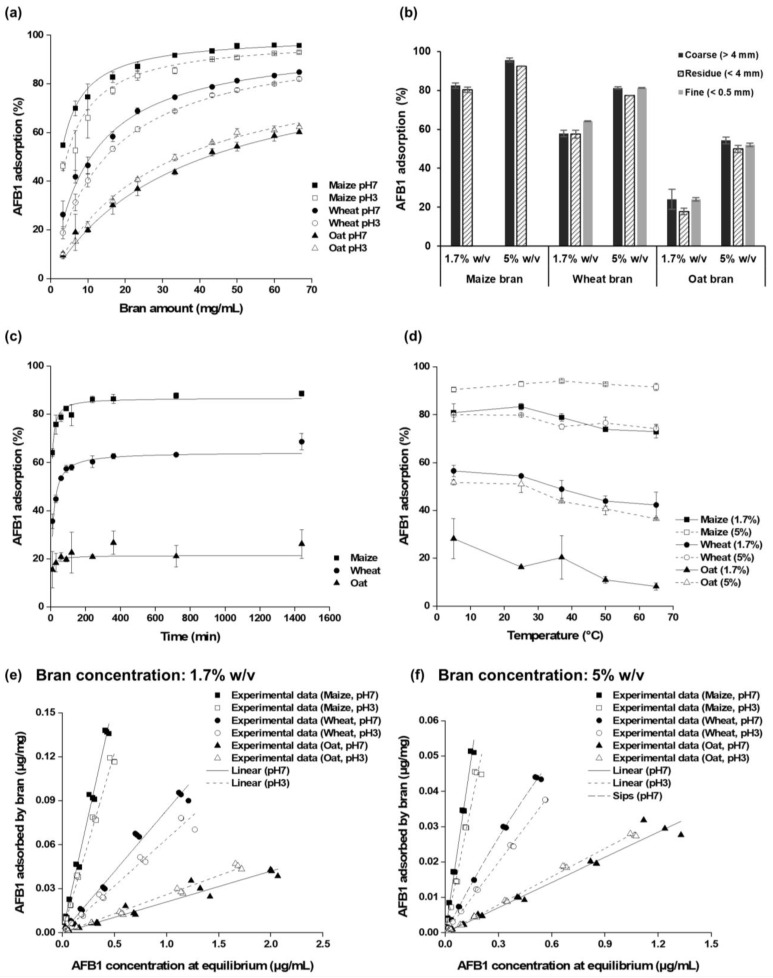
Effects of bran amount and pH (**a**), particle size (**b**), binding time (**c**), temperature (**d**), and AFB1 concentration (**e**,**f**) on AFB1 adsorption by maize, wheat, and oat bran. Equilibrium adsorption isotherms were obtained at constant temperature (25 °C) and pH (3 and 7) by a fixed amount of AFB1 (1 μg/mL) with an increasing bran amount (3–67 mg/mL corresponding to 0.3–6.7% *w*/*v*) (a) or by a fixed amount of bran at 17 mg/mL (1.7% *w*/*v*, (**e**)) and 50 mg/mL (5% *w*/*v*, (**f**)) with increasing AFB1 concentrations (0.05–5 μg/mL). Adsorption experiments were performed at pH 7 using 1.7% *w*/*v* or 5% *w*/*v* of bran amount and 1 μg/mL AFB1 concentration under 25 °C (**b**,**c**) or different temperatures (5–65 °C, (**d**)). The presented data are the mean ± standard deviation from three replicates (**a**–**d**).

The effects of bran amount and pH on AFB1 adsorption capacity were examined using different amounts of brans (0.3% to 6.7% *w*/*v*, corresponding to 3–67 mg/mL), each mixed with 1 μg/mL of AFB1 at both pH 3 and 7 (Figure 2a). AFB1 adsorption capacity improved with an increasing amount of bran. As bran amount increased from 0.3% to 6.7%, AFB1 adsorption ranged from 46.3% to 95.7% for maize bran, 18.8% to 84.8% for wheat bran, and 9.5% to 62.3% for oat bran. Greco et al. [18] reported the AFB1 adsorption capacities for agricultural by-products as follows: 6–27% for orange or lemon, 51–55% for pomegranate, 55% for stalks and leaves of the artichoke, 87% for almond hull, 83–94% for grape, and 100% for carobs. Under conditions similar to those examined in the study of Greco et al. [18] (1 μg/mL of AFB1, 10 mg/mL of adsorbent), maize and wheat bran reduced 74.6% and 46.5% of AFB1, respectively (Figure 2a). This indicates that different bioadsorbents can vary in their binding efficacy, likely due to variation in their surface chemistry. 

AFB1 is known to be absorbed in the duodenum, immediately after the stomach [27]. The human digestive system exhibits varying pH conditions in specific regions: the mouth (pH 5–7), stomach (pH 1–3), small intestine (pH 6–7.5), and large intestine (pH 5–7) [28]. These pH levels can directly influence the interaction between bran and mycotoxins. Thus, understanding the adsorption of AFB1 by different brans and also the stability of the bound complex at different pH levels is critical for comprehending the bioavailability of AFB1 in the gastrointestinal tract. Based on previous studies [18,29], pH 3 and 7 were selected as the representative pH of the gastrointestinal tract. In this study, both maize and wheat brans showed higher AFB1 adsorption at pH 7 than at pH 3, while oat bran adsorbed more AFB1 at pH 3 than at pH 7 (Figure 2a). The differences between pH 3 and 7, however, were not considerably distinct, as shown in Figure 2a, implying that AFB1 binding by bran is relatively stable under the acidic conditions present in the stomach. 

### 2.3. Effect of Particle Size on AFB1 Adsorption

AFB1 adsorption was evaluated using different particle sizes of three brans, with >4 mm classified as a coarse fraction, <4 mm as a residue fraction, and <500 μm as a fine fraction (Figure 2b). For all three brans, the coarse fractions (>4 mm) sequestered AFB1 more effectively than the residue fractions. Maximum adsorption was 95.6% for coarse maize bran at 5.0% *w*/*v* bran concentration. AFB1 adsorption levels of coarse and fine fractions were similar in wheat (both 81.3%) and oat (54.3% and 52.1%) at a higher bran concentration (5.0% *w*/*v*). However, when using a lower bran concentration (1.7% *w*/*v*), fine wheat bran showed significantly higher AFB1 adsorption (64.1%) compared to coarse wheat bran (57.9%). Reducing the particle size increased the bran surface area, which resulted in a higher AFB1 adsorption, observed at low bran concentration. Nevertheless, this phenomenon was not apparent at a higher bran concentration (5.0% *w*/*v*), presumably because, at this concentration, the available sites for AFB1 adsorption are already sufficient in both coarse and fine brans, and therefore the impact of particle size reduction is not observed. Previously, Avantaggiato et al. [19] also reported that grape pomace with a particle size under 500 μm (fine fraction) had a higher AFB1 adsorption capacity than grape pomace with a higher particle size.

### 2.4. Kinetics and Thermodynamics of AFB1 Adsorption

The kinetic change of AFB1 adsorption was determined by examining the binding time from 10 min to 24 h (Figure 2c). The adsorption rate of AFB1 was rapid between 10 and 60 min. Within 60 min, all three brans reached over half of the adsorption capacity and attained an equilibrium state after 90 min for maize bran and 12 h for wheat bran. Nonetheless, the adsorption rate slowed down after 60 min. The AFB1 adsorption by wheat bran increased slowly from 90 min (57.4%) to 24 h (68.7%). The adsorbed AFB1 by oat bran did not change significantly between 10 min and 24 h. In this study, an adsorption time of 90 min was selected, as it is comparable to food preparation or digestion time. 

AFB1 adsorption was studied at different temperatures (5, 25, 37, 50, and 65 °C). The experiments related to the temperature effect were performed using glass vials, due to the adsorption of AFB1 by the polypropylene microcentrifuge tubes at high temperatures (Supplementary Materials Figure S1). In general, the adsorbed AFB1 decreased at high temperatures (over 50 °C) for all brans (Figure 2d). The highest levels of AFB1 adsorption were at 37 °C for maize (94.1%) and at 5 °C for wheat and oat brans (79.9% and 51.7%, respectively) (Figure 2d). Additionally, when experiments at 65 °C were allowed to cool for 90 min rather than 15 min, AFB1 adsorption in maize bran did not increase, but it did in oat bran (1.7% *w*/*v*, 5.0% *w*/*v*) and wheat bran (5.0% *w*/*v*) (Supplementary Materials Figure S2). This finding confirmed that the lower temperatures were more conducive for adsorption of AFB1.

The differences in AFB1 adsorption capacities among maize, wheat, and oat brans can be attributed to the composition of the brans. As presented in Table 1, maize bran contained the highest levels of arabinose (14.1%), xylose (27.8%), and galactose (4.60%), leading to a total arabinoxylan content of approximately 38.6%. Conversely, oat bran predominately contained glucose (71.3%), attributed to the beta-glucan and possibly the starchy endosperm [15,16]. Wheat bran included glucose (37.7%), xylose (11.8%), arabinose (5.89%), galactose (0.87%), and arabinoxylan (17.1%). According to Roye et al. [30], arabinoxylan content, as reported in native and endosperm-depleted forms, is as follows: wheat bran (25% and 35.4%), oat bran (3.5% and 13.2%), and maize bran (10.7% and 42.7%). Interestingly, endosperm-depleted maize bran was mainly composed of the pericarp layer and contained pores, as evidenced by cryo-SEM and light microscopy images [30]. It is therefore likely that the maize bran used in this study was also composed of pericarp, with pores that enhanced its AFB1 binding capacity. To evaluate this hypothesis, wheat pericarp was carefully separated from wheat wholegrains and tested for AFB1 adsorption. The results showed that the wheat pericarp adsorbed 81.8% of AFB1 (1.7% *w*/*v*, 25 °C, pH 7), which was significantly higher than was observed in wheat bran (57.9%). Interestingly, this was similar to the adsorption of AFB1 by maize bran (82.5%). This demonstrated that the pericarp components in bran may be the central part of sequestering AFB1. 

### 2.5. Equilibrium Adsorption Isotherms

Various food-based adsorbents have been evaluated for mycotoxins using adsorption isotherm modeling [18,19,29,31]. Adsorption isotherms are crucial for understanding the interaction mechanism between an adsorbent and adsorbate at constant temperature [32,33,34]. In the present study, experiments on AFB1 adsorption isotherms were conducted using two bran amounts (1.7% and 5.0% *w*/*v*) at different pH levels (3 and 7) and a temperature of 25 °C while increasing AFB1 concentrations from 0.05 to 3 μg/mL. The linear regression model, following Henry’s law, and three non-linear regression models (Table 2, Figure 2e,f) were applied to assess the goodness of fit and calculate the adsorption parameters. All adsorption models demonstrated a good fit for the experimental data (R^2^ > 0.9786) (Table 2). 

Among the four isotherm models, the linear model was deemed the most suitable for all brans and conditions, except the wheat bran (5% *w*/*v*) at pH 7, where Sips model was the best fitted (Figure 2e,f). The favorability of AFB1 adsorption by brans was confirmed by separation factors (0.9968 < R_L_ < 1), which are close to 1, indicating linear adsorption. According to Wang and Gu [34], the linear model can explain the distribution of adsorbates between the solid and liquid phases. This process involves electrostatic, van der Waals, and hydrophobic interactions [34]. Due to the non-ionic property of AFB1, hydrophobic interactions are likely the primary mechanism for AFB1 adsorption by brans, as suggested by Avantaggiato et al. [19] and Wang and Guo [34]. The linear model describes monolayer adsorption at a low adsorbate concentration, which provides insight into the adsorption process between AFB1 and brans [32,34]. 

The maximum AFB1 adsorption (Ads_max_) and the bran amount required to achieve 50% AFB1 reduction (C_50_) were estimated using the Langmuir model [23,35], which correlated the AFB1 adsorption data with increasing bran amount (Figure 2a). The Langmuir model provided a good fit for the AFB1 adsorption results (R^2^ > 0.99) in all three brans (Figure 2a). The theoretical values of Ads_max_ and C_50_ for maize bran were 99.6% and 2.8 mg/mL at pH 7 and 98.8% and 4.3 mg/mL at pH 3; for wheat bran, 97.7% and 10.8 mg/mL at pH 7 and 99.9% and 14.8 mg/mL at pH 3; and for oat bran, 92.4% and 42.0 mg/mL at pH 7 and 91.7% and 34.6 mg/mL at pH 3. These estimated parameters were consistent with their experimental values across all three brans, as seen in Figure 2a. 

### 2.6. Effect of Solvent Volume on AFB1 Adsorption

The effect of solvent volume on AFB1 adsorption was investigated to identify the critical conditions for sequestering AFB1 using maize and wheat brans (Table 3). The AFB1 adsorption by maize bran was 82.4% and 68.6% in 1.5 mL and 3 mL solvent volume, respectively. After incubation, an additional 1.5 mL was added to the sample with an initial volume of 1.5 mL in order to dilute it to 3 mL. Despite this dilution, the AFB1 adsorption remained at 81.4%, which was comparable to the adsorption obtained with the sample containing 1.5 mL (82.4%). Similarly, wheat bran also showed higher AFB1 adsorption in the samples with 1.5 mL (54.1%) than the sample with 3 mL (39.6%) and the adsorption did not change (55.2%) when the sample with 1.5 mL was diluted to 3 mL. A lower initial solvent volume resulted in higher AFB1 adsorption for both maize and wheat brans, suggesting that increased interaction between AFB1 and bran in a low volume is crucial for enhancing the adsorption capacity. Interestingly, after the addition of an extra 1.5 mL, the AFB1 adsorption levels remained comparable to those with an initial solvent volume of 1.5 mL. This implies that once AFB1 is bound to the bran, it is held firmly and remains unaffected by subsequent changes in solvent volume. In contrast, charcoal adsorbed all AFB1 regardless of the initial volume or any changes in volume, demonstrating the differences in adsorption mechanisms between brans and charcoal. These results suggest that the bran surface adsorption of AFB1 is primarily driven by the frequency of their interactions. However, once bound, the bran effectively retains AFB1, possibly within surface pores [30]. 

These results provide insight into the potential of bran to reduce the bioavailability of AFB1. After ingestion, the volume of bolus increases in the gastrointestinal tract due to the secretion of biofluids in various parts of the digestive system [28]. The results therefore suggest that the AFB1–bran complex is stable despite the increasing volume in the gastrointestinal tract, thus preventing the absorption of AFB1 and consequently increasing its excretion.

## 3. Conclusions 

This study investigated the efficacy of cereal brans—specifically, maize, wheat, and oat brans—in adsorbing AFB1 and FMB1, as well as examined the mechanism by which brans capture AFB1. The results confirmed the potential of cereal brans to function as AFB1 adsorbents effectively and naturally. Maize bran demonstrated the highest AFB1 adsorption capacity, followed by wheat and oat brans. This research indicated that the primary site of adsorption in bran is the pericarp, which was further supported by the higher adsorption ability of wheat pericarp. AFB1 adsorption was marginally higher at pH 7 than at pH 3 for both maize and wheat bran, and it decreased above 37 °C for all brans. Interestingly, a lower initial volume was found to be critical for maximizing the adsorption capability, suggesting that the surface structure of bran, which likely contains pores, requires sufficient time and interaction to capture AFB1. In general, the best-fitted equilibrium isotherm model for the adsorption process between bran and AFB1 was the linear model, implying that the adsorption tends to occur at lower concentrations under the electrostatic, van der Waals, and hydrophobic interactions. These findings demonstrate that brans can efficiently interact with AFB1. Further studies are necessary to confirm the reduction in AFB1 bioavailability by brans through *in vivo* experiments. 

## 4. Materials and Methods

### 4.1. Chemicals and Materials

Aflatoxin B1 (AFB1), fumonisin B1 (FMB1), activated charcoal, phosphate-buffered saline (tablet form, PBS), and sorbitol were purchased from Sigma (Saint Louis, MO, USA). Acetyl chloride and trimethylsilyl chloride (TMSCl) were obtained from Fluka (Ballwin, MO, USA). Internal standards of ^13^C_34_-FMB1 and QuEChERS (MgSO_4_ 300 mg, C_18_ 50 mg) were purchased from LGC Standards GmbH (Wesel, Germany) and BEKOlut (Hauptstuhl, Germany), respectively. Acetonitrile (LC-MS grade, ACN), methanol (HPLC grade), acetone (HPLC grade), and heptane (HPLC grade) were purchased from Honeywell Riedel de Haën (Seelze, Germany). Formic acid (LC-MS grade, FA) was obtained from VWR Chemical (Belgium). Citrate acid monohydrate, disodium hydrogen phosphate, ammonium acetate (LC-MS grade), acetic acid (LC-MS grade), pyridine, bis(trimethylsilyl)trifluoroacetamide (BSTFA), and the monosaccharide standards, including L-arabinose, D-xylose, D-glucose, and D-galactose, were purchased from Merck KGaA (Germany). The stock solutions of AFB1 (1 mg/mL) and FMB1 (0.5 mg/mL) were prepared in ACN and 50% ACN, respectively, and stored in amber glass vials at −20 °C. Citrate-phosphate buffer (CPB, 0.1 M, pH 3 and pH 7) was prepared by mixing 0.1 M citric acid solution and 0.2 M disodium hydrogen phosphate solution. Ammonium formate buffer (1 mM, pH 3) was utilized for the FMB1 adsorption experiment. Milli-Q water (Millipore, Saint Louis, MO, USA) was employed throughout the study.

Wheat bran (coarse and fine) and oat bran (coarse, unheated) were obtained from Fazer Mill (Lahti, Finland) and Mäkelän luomutila (Ryttylä, Finland), respectively. Maize bran was provided by the Institut de Recherche en Sciences Appliquées et Technologies/Département Technologie Alimentaire (IRSAT/DTA, Ouagadougou, Burkina Faso). All bran samples were stored at 5 °C. Fine particle size of the oat bran was prepared by milling and homogenizing oat bran through a 0.5 mm sieve using a Mortar Grinder RM 200 (RetschGmbH, Haan, Germany) and subsequently stored at −20 °C. Before using the commercially available brans, the presence of AFB1 and FMB1 was confirmed in maize, wheat, and oat bran. Both wheat and oat bran were free of AFB1 and FMB1. Maize bran, however, contained FMB1. Therefore, this was accounted for by subtracting the level of fumonisin B1 in the blank from its level in the samples.

Wheat pericarp was prepared manually from wholegrain wheat (Fazer Mill, Finland). Briefly, individual wholegrain wheat was soaked with a minimal amount of water (~20 μL) in a 96-well plate. Subsequently, the pericarp was gently detached from the wholegrain wheat with tweezers. The pericarp was dried at room temperature and preserved in a desiccator. All brans and the wheat pericarp utilized in this study are shown in Supplementary Materials Figure S3.

### 4.2. AFB1 and FMB1 Adsorption Experiments

Coarse bran or pericarp fraction (25 mg, 1.7% *w*/*v*) in 1.5 mL buffer containing 1 μg/mL AFB1 (0.1 M CPB, pH 3 or 7) was used to determine AFB1 adsorption capacity. The mixture was vortexed and incubated in a shaker (Stuart S150, BIBBY STERILIN LTD, Staffordshire, UK) at 200 rpm speed for 90 min at a constant temperature (25 °C). The tube was left for 15 min at room temperature before the supernatant was transferred to a new microcentrifuge tube. After centrifugation (16,100× *g* for 10 min), the supernatant of AFB1 samples was filtered through an Acrodisc syringe filter (0.2 μm, wwPTFE, Pall Corporation, NY, USA). FMB1 (5 μg/mL) adsorption by bran (25 mg, 1.7% *w*/*v*) was evaluated in water (pH 6.5) and 1 mM ammonium formate buffer (pH 3). CPB was selected for the AFB1 adsorption experiments due to its wide pH coverage (pH 2.6 to 7.6). Preliminary tests confirmed that there was no difference between utilization of 0.1 M CPB (pH 7) or PBS (pH 7.4), which is widely used in other studies [18,19]. For the FMB1 adsorption experiments, 1 mM ammonium formate (pH 3) and Milli-Q water (pH 6.5) were utilized because the analysis method involving liquid chromatography–mass spectrometry (LC-MS) is not compatible with buffers containing sodium or potassium salts. 

After incubation, the samples were centrifuged, and 1 mL of the supernatant was spiked with 20 μL of ^13^C_34_-labelled FMB1 working solution (5.1 μg/mL in 50% ACN) as an internal standard and mixed thoroughly with 1 mL of ACN (1% FA). After cleaning up with QuEChERS, the samples were centrifuged (16,100× *g* for 10 min) and the supernatant was collected for analysis. A negative control was prepared in buffer or water containing 1 μg/mL AFB1 or 5 μg/mL FMB1 without bran. Activated charcoal was treated in a similar way and was used as a positive control. All adsorption studies were carried out in triplicate.

#### Factors Influencing AFB1 Adsorption

The effects of bran amount, pH, bran particle size, adsorption time, temperature, and solvent volume on AFB1 adsorption were evaluated. Firstly, the effects of bran amount and pH on AFB1 adsorption were investigated simultaneously at different pH values (3 and 7) using a constant AFB1 concentration (1 μg/mL) with various bran amounts (0.3–6.7% *w*/*v* corresponding to 3.0–67 mg/mL). For the effect of particle size, three fractions were examined: over 4 mm (coarse), under 4 mm (residue), and under 0.5 mm (fine). The coarse and residue parts were separated by sieving (4 mm) the brans. In the case of fine fractions, fine wheat bran was purchased by Fazer Mill (Finland) and the oat bran was milled as indicated earlier. Each fraction was treated at two different bran amounts (1.7% and 5% *w*/*v* corresponding to 17 and 50 mg/mL) in buffer (0.1 M CPB, pH 7) containing 1 μg/mL AFB1. The effect of the binding time on the AFB1 adsorption was performed as previously described for maize, wheat, and oat brans (1.7% *w*/*v*) in 0.1 M CPB (pH 7) containing 1 μg/mL AFB1 at 25 °C. However, the samples were incubated for 10 min to 24 h (nine time points) before analyzing free AFB1. The effect of temperature was assessed at pH 7 using two different bran amounts (1.7% and 5% *w*/*v*) and a fixed AFB1 concentration (1 μg/mL) in 4 mL glass vials instead of microcentrifuge tubes. The samples were incubated at 5, 25, 37, 50, and 60 °C for 90 min. The adsorption process was conducted as previously described. Moreover, following the incubation, the glass vials were allowed to stand for 90 min, as opposed to the typical 15 min used in other experiments, to evaluate any differences in AFB1 adsorption due to varying cooling time.

To investigate the effect of solvent volume on AFB1 adsorption, maize and wheat brans were evaluated as previously described but with a final volume of 3 mL instead of 1.5 mL. In the first experimental setup, an additional 1.5 mL buffer was added after the incubation period with 1.5 mL, and in the second experimental setup, the adsorption was carried out with bran (25 mg) in 3 mL of buffer containing AFB1 (1 μg/mL). 

Adsorption isotherms were obtained at constant temperature (25 °C) and pH values of 3 and 7 (0.1 M CPB) using a fixed amount of bran (1.7% and 5% *w*/*v*) with varying AFB1 concentrations (0.05–5 μg/mL). These isotherms were used to calculate the parameters related to AFB1 adsorption by bran (Table 4).

### 4.3. Monosaccharide Analysis in Brans

The monosaccharide composition of the brans was analyzed after acidic methanolysis according to Sundberg et al. [36] and Van Craeyveld et al. [37]. For the analysis, 10 mg of cereal bran and standard monosaccharides were weighed into pear-shaped flasks and dried in a Speed-Vac Plus equipped with a Universal Vacuum System Plus with Vapornet UVS400 (Savant Instruments, Inc., Austin, TX, USA) at 30 °C for 30 min. The samples and standards were treated with acid methanolysis reagent (2 M HCl in dry methanol) at 100 °C for 3 h. The monosaccharides were analyzed after trimethylsilylation by a gas chromatograph (Hewlett-Packard 5890 series II) equipped with a flame ionization detector using a splitless injection mode (Hewlett-Packard, USA) connected to an HP 5 capillary column (30 m, 0.32 mm i.d., 0.25 μm film thickness; Agilent Technologies, Santa Clara, CA, USA). The amounts of the monosaccharides were calculated as anhydrous sugars (0.88 for pentoses, 0.90 for hexoses). 

### 4.4. AFB1 Analysis Using UPLC-FLD

For the AFB1 analysis, 20 μL of ACN was added to 180 μL of sample or standard solution to match the initial condition of the mobile phase. AFB1 was analyzed by ultra-performance liquid chromatography with a fluorescence detector (Acquity UPLC class, Waters, Milford, MA, USA). Fluorescence detection of AFB1 was performed using excitation and emission wavelengths of 365 nm and 435 nm, respectively. Chromatographic separation was acquired by an Acquity UPLC BEH C18 column (2.1 × 100 mm, 1.7 μm) connected to an Acquity UPLC BEH C18 VanGuard^TM^ pre-column (2.1 × 5 mm, 1.7 μm) from Waters at a constant temperature (40 °C). The mobile phases A and B were 0.1% acetic acid in Milli-Q water and acetonitrile, respectively, at a constant flow rate (0.4 mL/min). The gradient conditions were as follows: 10% B from 0 to 0.5 min, 10–70% B from 0.5 to 6.0 min, 70% B from 6.0 to 6.5 min, 70–10% B from 6.5 to 7.0 min, and 10% B from 7.0 to 9.0 min. The injection volume was 5 μL, and the autosampler temperature was set at 10 °C. 

The AFB1 concentration range was 0.02–5 μg/mL for the equilibrium adsorption experiment and 0.02–2 μg/mL for the other experiments. Calibration solutions were prepared in 0.1 CPB (pH 3 or 7). The integration of peaks was carried out using Empower 2 (Waters). 

### 4.5. FMB1 Analysis Using UPLC-MS

FMB1 was analyzed using ultra-performance liquid chromatography (Acquity UPLC class, Waters) with quadrupole time-of-flight (Synapt G2-si, Waters). The columns mentioned in the AFB1 analysis were used under a constant mobile phase rate of 0.3 mL/min. The mobile phase consisted of solution A (1 mM ammonium acetate, 0.5% acetic acid, and 0.1% FA in Milli-Q water) and solution B (0.5% acetic acid and 0.1% FA in ACN). The gradient conditions were as follows: 5% B from 0 to 0.5 min, 5–50% B from 0.5 to 4.5 min, 50–100% B from 4.5 to 5.5 min, 100% B from 5.5 to 6.5 min, 100–5% B from 6.5 to 6.6 min, and 5% B from 6.6 to 9 min. The injection volume was 5 μL. Column oven temperature (40 °C) and autosampler temperature (10 °C) were used in the UPLC system. The conditions of the ESI source in positive and sensitivity mode were as follows: source voltage, 0.5 kV; source temperature, 120 °C; desolvation temperature, 600 °C; cone gas, 50 L/h; cone voltage, 10 V; desolvation gas, 1000 L/h; and nebulizer gas, 6 bar. The multiple monitoring transitions of FMB1 and FMB1 IS were established as 722.39 → 334.40*/352.40 and 756.40 → 374.37*/356.36, respectively, under the collision energy ramp from 30 to 40 eV. The quantifier (*) and qualifier ions were selected based on the most and second-most intense transitions from the parent ion to the fragment ion and also used in the literature [38]. 

FMB1 calibration solutions were prepared in 1 mL of water (pH 6.5) or 1 mM ammonium formate (pH 3) with a range of 0.01–7 μg/mL. The same procedure using QuEChERS was applied to the calibration solutions spiked with the FMB1 IS. The area ratios of FMB1 and FMB1 IS were used to calculate FMB1 concentration using Masslynx and Quanlynx (Waters). 

### 4.6. Adsorption Calculation and Model Fitting

The amount of adsorbed AFB1 was calculated based on the difference in AFB1 concentrations between the supernatant of the negative control (without brans) and the supernatant of the experimental tubes containing brans or active charcoal. AFB1 adsorption capacity was calculated using the following equation, as described by Avantaggiato et al. [19]:

*q_e_* = (*C_i_* − *C_e_*) × V/m, (1)

where *q_e_* represents the quantity of AFB1 adsorbed per milligram of bran (μg/mg), *C_i_* is the concentration of AFB1 in the supernatants of the negative control without bran (μg/mL), *C_e_* is the residual AFB1 concentration in the supernatant of the experimental samples with brans at equilibrium (μg/mL), V is the volume of the solution (mL), and m is the mass of bran (mg).

AFB1 adsorption was calculated using Excel (Microsoft 365), and statistical analyses including *t*-test, ANOVA, and post hoc tests (Tukey HSD) were performed using IBM SPSS Statistics (IBM Corp., Armonk, NY, USA). Adsorption isotherms were fitted by plotting the amount of AFB1 adsorbed per mass unit of bran (*q_e_*) against the concentration of AFB1 (*C_e_*) in the supernatant under the equilibrium conditions using Origin 2021b (OriginLab Corporation, Northampton, MA, USA). Both linear and non-linear models, including Langmuir, Freundlich, and Sips, were applied to the experimental adsorption data, and the separation factor (*R_L_*) was also calculated from the Langmuir model (Table 4) [18,19,34]. The coefficient of determination (*R*^2^) was used to confirm the fitness of the model.

## Figures and Tables

**Table 1:** Carbohydrate profiles of maize, wheat, and oat brans (g/100 g of the fraction, dry weight basis) presented as average ± standard deviation.

Cereal Bran	Carbohydrate Composition (Mean ± SD, %)				
	Arabinose	Xylose	Galactose	Glucose	Arabinoxylan ^†^
Maize	14.1 ± 3.0	27.8 ± 5.4	4.6 ± 0.6	12.5 ± 0.9	38.6 ± 8.0
Wheat	5.9 ± 1.1	11.8 ± 1.4	0.87 ± 0.06	37.7 ± 2.7	17.1 ± 2.5
Oat	1.16 ± 0.03	1.44 ± 0.02	0.57 ± 0.08	71.3 ± 2.7	2.21 ± 0.08

^†^ Arabinoxylan = (Arabinose − 0.7 × Galactose) + Xylose.

**Table 2:** Isotherm model parameters for the adsorption of AFB1 by maize, wheat, and oat brans (1.7% and 5.0% *w*/*v*) at different pH values.

Model /Parameters	Bran Concentration											
	1.7% *w*/*v*						5.0% *w*/*v*					
	Maize Bran		Wheat Bran		Oat Bran		Maize Bran		Wheat Bran		Oat Bran	
	pH 7	pH 3	pH 7	pH 3	pH 7	pH 3	pH 7	pH 3	pH 7	pH 3	pH 7	pH 3
Linear												
*K* (mL/mg)	0.3185	0.2452	0.0834	0.0638	0.0210	0.0260	0.3371	0.2467	0.0859	0.0664	0.0237	0.0265
R^2^	0.9951	0.9963	0.9929	0.9919	0.9780	0.9949	0.9960	0.9921	0.9979	0.9994	0.9886	0.9986
Langmuir												
*q_m_* (μg/mg)	3.4216	1.0344	0.7289	0.8121	0.2371	59.0617	0.6173	1.0212	0.2970	4.3319	0.5291	0.4553
*K_L_* (mL/μg)	0.0963	0.2624	0.1286	0.0853	0.1044	0.0004	0.5852	0.2509	0.3309	0.0154	0.0470	0.0613
R^2^	0.9909	0.9945	0.9888	0.9858	0.9617	0.9907	0.9934	0.9855	0.9986	0.9989	0.9788	0.9978
Freundlich												
*K_F_* (mL^1/n^ μg^1−1/n^/mg)	0.3126	0.2329	0.0829	0.0637	0.0219	0.0253	0.3106	0.2490	0.0800	0.0656	0.0237	0.0264
*1*/*n*	0.9824	0.9464	0.9424	0.9581	0.9158	1.0951	0.9623	1.0047	0.9208	0.9841	0.9832	0.9774
R^2^	0.9908	0.9940	0.9877	0.9854	0.9604	0.9931	0.9931	0.9852	0.9982	0.9990	0.9786	0.9975
Sips												
*q_ms_* (μg/mg)	1.2661	0.4603	0.2295	0.3151	0.1022	8.9526	0.2902	0.0982	0.2546	7.8490	0.1136	0.1110
*K_S_* (mL^ns^/μg^ns^)	0.2901	0.7519	0.5769	0.2560	0.2894	0.0028	1.5528	9.0377	0.4015	0.0084	0.2683	0.3107
*1*/*n_s_*	1.0434	1.1024	1.2525	1.1072	1.1938	1.0979	1.0627	1.4011	1.0167	0.9866	1.1535	1.1373
R^2^	0.9909	0.9948	0.9903	0.9861	0.9626	0.9931	0.9935	0.9896	0.9986	0.9990	0.9795	0.9983

**Table 3:** Effect of solvent volume on aflatoxin B1 adsorption by maize and wheat brans.

Initial Volume (mL)	Added Volume (mL)	Settling Time (min)	Aflatoxin B1 Adsorption ^†^ (Mean ± SD, %)		
			Maize Bran	Wheat Bran	Charcoal
1.5	-	15	82.4 ± 2.8 ^a^	54.1 ± 3.7 ^a^	100
1.5	1.5	15	81.4 ± 1.4 ^a^	55.2 ± 6.9 ^a^	100
3.0	-	15	68.6 ± 1.2 ^b^	39.6 ± 1.2 ^b^	100

^†^ Values labelled with the same letters in a column are not significantly different (*p* < 0.05).

**Table 4:** Isotherm models and equations used to analyze aflatoxin B1 adsorption data [18,19,34].

Model	Equation	Description
Linear (Henry’s law)	$q_{e}={KC}_{e}$	*q_e_* is the adsorbed amount at equilibrium (μg/mg) *C_e_* is the adsorbate concentration at equilibrium (μg/mL) *K* is the partition coefficient (mL/mg)
Langmuir	$q_{e}=\frac{{q_{m}K}_{L}C_{e}}{1+K_{L}C_{e}}$	*q_m_* is the maximum adsorption capacity (μg/mg) *K_L_* is the ratio of the adsorption rate and desorption rate (mL/μg)
	$R_{L}=\frac{1}{1+K_{L}C_{0}}$	*R_L_* ^†^ is the separation factor *C*_0_ is the low initial adsorbate concentration (μg/mL)
Freundlich	$q_{e}=K_{F}C_{e}^{1/n}$	*K_F_* (mL^1/n^ μg^1−1/n^/mg) and *n* are the Freundlich constants
Sips ^‡^	$q_{e}=\frac{q_{ms}K_{S}C_{e}^{n_{s}}}{1+K_{S}C_{e}^{n_{s}}}$	*q_ms_* is the maximum adsorbed amount (μg/mg) *K_s_* (mL^ns^/μg^ns^) and *n_s_* are the Sips constants

^†^ Adsorption is unfavorable (*R_L_* > 1), linear (*R_L_* = 1), or favorable (*R_L_* < 1). ^‡^ The Sips model is a hybrid model containing the Langmuir model and the Freundlich model. It can change to the Langmuir model (*n_s_* = 1) and the Freundlich model (low *C*_0_).

## Data Availability

All data are presented in the article.

## Supplementary Materials

The following supporting information can be downloaded at: https://www.mdpi.com/article/10.3390/toxins16070288/s1, Figure S1: Effect of containers on AFB1 adsorption at different temperatures; Figure S2: Effect of cooling time on AFB1 adsorption; Figure S3: Maize, wheat and oat bran and wheat pericarp utilized in this study.
