# Supplementary material for: Efficacy of Aflatoxin B1 and Fumonisin B1 Adsorption by Maize, Wheat, and Oat Bran
Source: Toxins (Basel). 2024 Jun 25;16(7):288. doi: 10.3390/toxins16070288 (PMC11281723; doi:10.3390/toxins16070288)
Supplement: Supplementary file 1 [file toxins-16-00288-s001.zip › toxins-3003176-supplementary.pdf]

toxins

MDPI

Supplementary Materials: Efficacy of Aflatoxin B1 and Fumonisin B1 Adsorption by Maize, Wheat, and Oat Bran

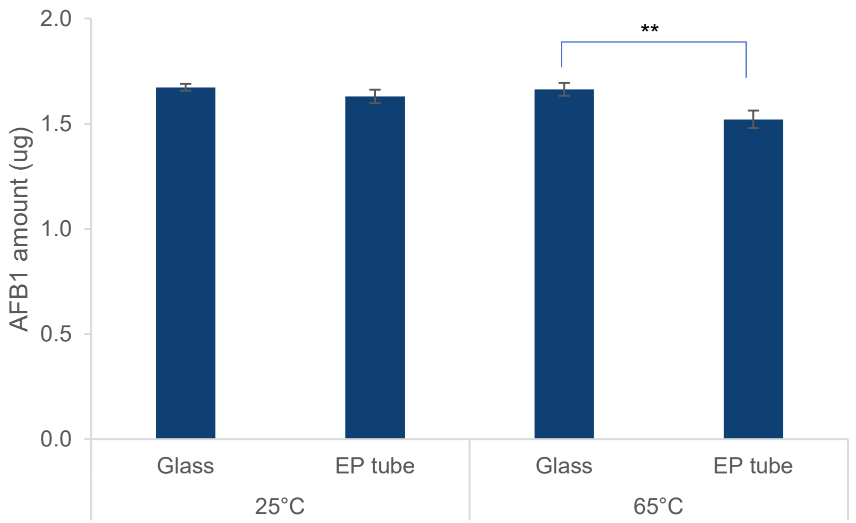

Figure S1. Effect of containers on AFB1 adsorption at different temperatures. Adsorption experiments were performed at pH 7 using 1 μg/mL AFB1 concentration under 25 °C or 65 °C for 90 min. The data presented are the mean ± standard deviation from three replicates. Statistical significance is denoted by asterisks (*** $p < 0.001$, ** $p < 0.01$, and * $p < 0.05$).

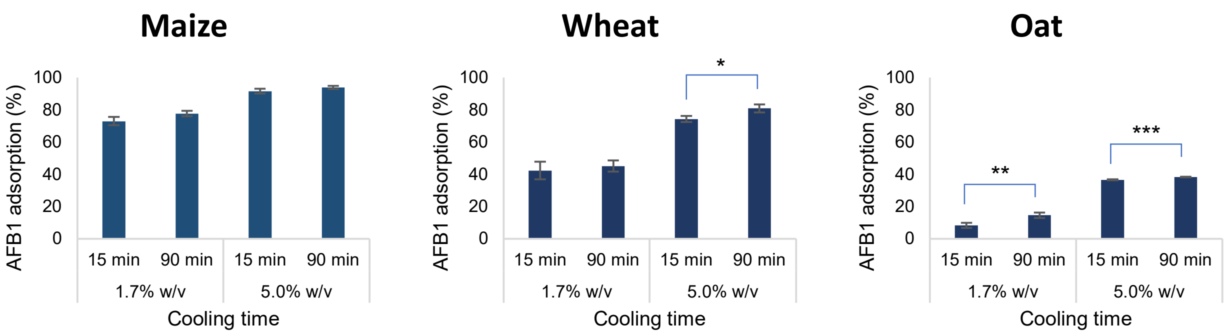

Figure S2. Effect of cooling time on AFB1 adsorption. Adsorption experiments were performed at pH 7 using 1.7% w/v or 5% w/v of bran amount and 1 μg/mL AFB1 concentration at 65 °C for 90 min, followed by cooling for 15 min and 90 min. The presented data are the mean ± standard deviation from three replicates. Statistical significance is denoted by asterisks (*** $p < 0.001$, ** $p < 0.01$, and * $p < 0.05$).

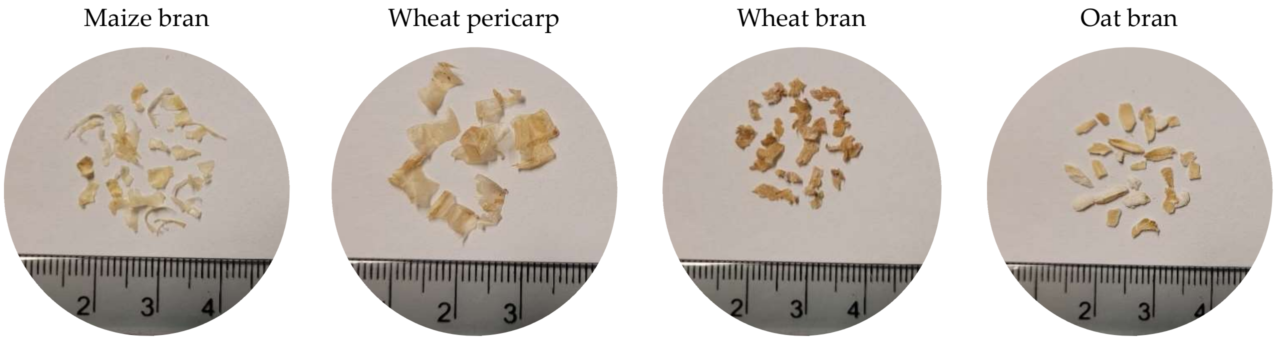

Figure S3. Maize, wheat and oat bran and wheat pericarp utilized in this study.
